# Tadpole-transporting frogs use stagnant water odor to find pools in the rainforest

**DOI:** 10.1242/jeb.243122

**Published:** 2021-11-05

**Authors:** Shirley J. Serrano-Rojas, Andrius Pašukonis

**Affiliations:** 1 Department of Biology, Stanford University, 371 Jane Stanford Way, Stanford, CA 94305, USA; 2 Universidad Nacional de San Antonio Abad del Cusco (UNSAAC), Cusco 08000, Perú; 3 CEFE, Univ. Montpellier, CNRS, EPHE, IRD, Montpellier, 34090, France

**Keywords:** Tropical amphibians, Water finding, Navigation, Frog olfaction, Chemical cues, Reproductive strategy

## Abstract

Breeding sites are often a limited and ephemeral resource for rainforest frogs. This resource limitation has driven the evolution of diverse reproductive strategies that increase offspring survival. For example, poison frogs shuttle their tadpoles from terrestrial clutches to aquatic rearing sites, using various cues to assess pool suitability. Yet, how frogs find new pools is unknown. We tested the role of odor cues in the process of finding tadpole deposition sites by the poison frog *Allobates femoralis*. We created 60 artificial pools grouped into three conditions: stagnant water, tadpole water and clean water control. Fifteen pools were discovered within 6 days, with more tadpoles and more frogs directly observed at pools with stagnant odor cues. Our findings suggest that frogs use odor cues associated with stagnant water for the initial discovery of new breeding pools. These cues may be good indicators of pool stability and increased likelihood of tadpole survival.

## Abstract (precis)

**Summary:** Amphibians rely on water for reproduction; however, very little is known on how amphibians find water bodies. Experiments in *Allobates femoralis* suggest that frogs use stagnant odor cues to find breeding pools.

## INTRODUCTION

Most amphibians rely on water to successfully complete their life cycle (Wells, 2007). Therefore, the ability to find suitable water bodies is vital for amphibian survival (Duellman and Trueb, 1986; Wells, 2007). Most temperate-region amphibians use relatively stable streams and ponds at known localities and under predictable seasonal climatic conditions (Pernetta and Geltsch, 2007; Pittman et al., 2014; Sinsch, 2014). Chemical stimuli learned during larval development (i.e. natal site imprinting) and/or the ability to perceive long-distance cues from the breeding sites in part explain the ability of temperate-region amphibians to return to their natal ponds and find new ones (reviewed in Ogurtsov, 2004; Ogurtsov and Bastakov, 2001; Sinsch, 1990, 2006). In tropical environments, water is often abundant, but suitable breeding sites are rare because of high predator pressure (McKeon and Summers, 2013; Schulte et al., 2013) and high desiccation risk owing to high evaporation rates (Poelman and Dicke, 2007; Schulte and Lötters, 2013). How amphibians find suitable water bodies in complex and unpredictable environments such as tropical rainforest is still largely unknown.

Tropical amphibians evolved to use a variety of aquatic ephemeral sites for reproduction, such as small standing-water bodies inside plant structures (i.e. phytotelmata) and temporarily flooded depressions, reducing the risk of predation by large predators present in permanent ponds or streams (Summers and McKeon, 2004). The quality of these small pools is variable (Fouilloux et al., 2021; Rudolf and Rödel, 2005) and the seasonality of tropical regions has a strong impact on breeding site availability (Bertoluci and Rodrigues, 2002; Gottsberger and Gruber, 2004). In response to the uncertainty of these breeding resources, frogs have presumably evolved strategies allowing them to find, assess and choose the appropriate rearing sites that maximize offspring survival (McKeon and Summers, 2013; Poelman et al., 2013; Ringler et al., 2013, 2018).

Poison frogs, for example, have evolved parental care strategies, such as transporting tadpoles from terrestrial oviposition sites to widely dispersed ephemeral tadpole-rearing pools (Pašukonis et al., 2019; Wells, 2007). This complex spatial behavior evolved along with the behavioral plasticity in tadpole deposition strategies that balance the benefits and risks involved. Pool choices are influenced by an interaction of many factors, such as physical and chemical characteristics (Brown et al., 2008; Fouilloux et al., 2021; McKeon and Summers, 2013; Poelman and Dicke, 2007; Poelman et al., 2013), distance to the territory (Erich et al., 2015; Pašukonis et al., 2019; Ringler et al., 2013, 2018), predator presence, type and abundance (McKeon and Summers, 2013; Ringler et al., 2018; Schulte et al., 2013; Von May et al., 2009), conspecific presence (McKeon and Summers, 2013; Rojas, 2014; Schulte et al., 2013), and desiccation risks (Poelman and Dicke, 2007; Schulte and Lötters, 2013). Tadpole-transporting parents can use chemical cues to detect the presence of predators or conspecific tadpoles (Rojas, 2014; Schulte and Lötters, 2013, 2014; Schulte et al., 2011, 2015). Recent findings have also shown that some poison frogs use spatial memory to find and efficiently exploit pools tens to hundreds of meters away from the territory (Beck et al., 2017; Erich et al., 2015; Pašukonis et al., 2016, 2019; Ringler et al., 2013), but how frogs find these pools in the first place is still unknown.

A study by Pašukonis et al. (2016) found that tadpole-carrying brilliant-thighed poison frogs, *Allobates femoralis*, were attracted to out-of-reach pools containing water with high concentrations of conspecific tadpoles, and proposed that conspecific olfactory cues may facilitate the initial discovery of new deposition sites. However, an alternative hypothesis is that the pool water itself (and not the tadpoles in it) was responsible for attracting the frogs. To complete tadpole development, *A. femoralis* needs small pools of standing water that do not dry out for 2 to 3 months. Therefore, cues associated with standing water, such as decomposing leaf odor, that are indicators of pool stability may also provide a cue for finding new tadpole rearing sites.

To test these hypotheses, we first conducted a pilot study and found that pools with stagnant water or decomposing leaves were rapidly occupied by *A. femoralis* while pools with clean water and conspecific tadpoles remained empty. Based on these preliminary observations, we designed a large-scale field experiment to test the role of odor cues associated with standing water in the process of finding new pools by *A. femoralis*.

## MATERIALS AND METHODS

### Study site and system

The study was carried out between 31 January and 6 March 2020 in the lowland rainforest near the field camp Saut Pararé (4°02′N, 52°41′W) at the Nouragues Ecological Research Station in the Nature Reserve Les Nouragues, French Guiana.

**Fig. 1.**
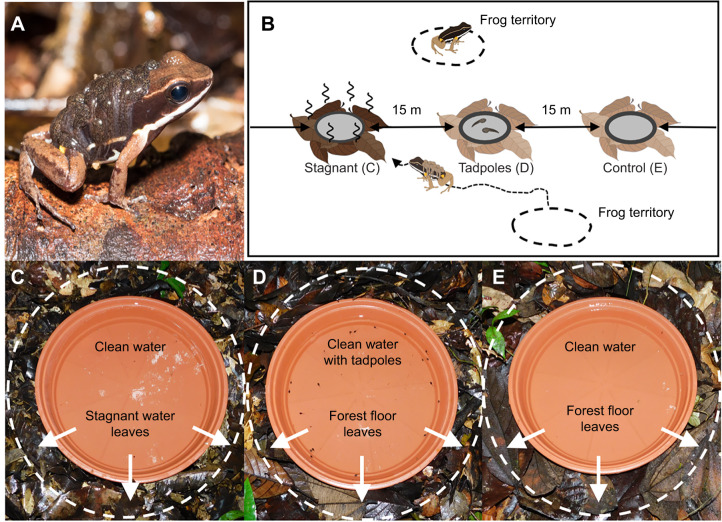
**Study species and experimental setup.** (A) Photograph of a male *Allobates femoralis* transporting tadpoles on his back. (B) Schematic representation of the experimental setup. Photographs of each experimental pool condition are shown for (C) stagnant water cues (stagnant water condition), (D) conspecific tadpole cues (tadpole water condition) and (E) clean water (control condition). The dashed white circle and white arrows highlight the leaf litter surrounding the pools, which was either soaked (C) or not (D,E) in stagnant water for 2 weeks prior to setting up the transect. The same clean water was used in all three conditions, with *A. femoralis* tadpoles added to the tadpole condition (D).

*Allobates femoralis* (Boulenger 1884) is a small diurnal leaf-litter frog distributed throughout the Amazonian basin and the Guiana Shield (Amézquita et al., 2009, 2017). Males vocally advertise territories to attract females and mating takes place inside the male's territory, where clutches of approximately 20 eggs are laid in the leaf litter (Ringler et al., 2009; Roithmair, 1992; Stückler et al., 2019). Tadpole transport takes place after 15–20 days of larval development when primarily males carry 1 to 25 tadpoles to small pools (Ringler et al., 2013) (Fig. 1A). Frogs travel tens to hundreds of meters to deposit their tadpoles, spread them across several pools (Erich et al., 2015) and readily use artificial pools (Ringler et al., 2015). *Allobates femoralis* tadpoles are omnivorous but not predatory.

### Ethics

The study was approved by the scientific committee of the Nouragues Ecological Research Station and the Institutional Animal Care and Use Committee of Stanford University (protocol ID 33211). We strictly adhered to the current US, French and European Union law, and followed the Association for the Study of Animal Behaviour's (ASAB) Guidelines for the use of live animals in teaching and research (ASAB, 2018).

### Experimental design

We placed three types of artificial pool conditions in the field (Fig. 1B–E): pools with stagnant water cues (stagnant water condition), pools with conspecific tadpole cues (tadpole water condition) and clean water (control condition). To produce the stagnant water odor, we used decaying leaf litter fermented in river water. Based on preliminary experiments, 5 liters of leaf litter were soaked for 2 weeks inside a mesh-covered plastic outdoor container with 40 liters of river water. This decomposing leaf litter was then used to prepare experimental pools.

Each artificial pool consisted of a brown plastic plant saucer (diameter 35 cm) filled with 2 liters of untreated water from a shallow groundwater well from the field station. For the stagnant water condition, we surrounded the pool with 2 liters of wet decomposing leaf litter (Fig. 1C). For the tadpole water condition, we added ∼18 (±4) tadpoles of *A. femoralis* in different developmental stages collected from various pools in the area. For the latter and the control condition, we surrounded the pool with 2 liters of leaf litter from the forest floor, making them visually similar to the stagnant water condition (Fig. 1D,E). Therefore, in the final setup, the stagnant water pools only differed from the control pools in that the leaf litter immediately surrounding the pool was soaked in standing water for 2 weeks prior to the start of the experiment.

We set four non-simultaneous linear transects in areas of high frog density, separated from each other by at least 50 m. Each transect had 15 pools separated by 15 m (Fig. 1B). Transects were located parallel to established trails, but pools were placed at least 2 m away from the trails. The order of the experimental pool conditions was randomized between every three consecutive pools within the transect.

### Data collection

After the setup day, we recorded the number of new *A. femoralis* tadpoles deposited in each pool at end of the day (17:30–19:00 h) for 6 days. In addition, pools were checked every morning during the period of high tadpole transport activity (Beck et al., 2017; Ringler et al., 2013) to record the presence of *A. femoralis* visiting the pools. Each tadpole does not represent an independent sample of pool discovery because a single frog can deposit up to 25 tadpoles at once (Ringler et al., 2013) and the same frog can transport several times to the same pool. As we cannot assess how many independent depositions happened in each pool, the only independent sample in our data is the presence or absence of tadpoles (probability of tadpole deposition). However, because frogs often split tadpoles between multiple pools (Beck et al., 2017; Erich et al., 2015), the number of tadpoles is a good indicator of pool preference among the pools that were found. Therefore, we use both the probability of tadpole deposition and the tadpole number in our data analysis, with slightly different interpretations.

Finally, we used capture–recapture data to obtain some information on distances from which *A. femoralis* discover new pools. Male territory locations were determined as part of a long-term capture–recapture study and following previously established methods (Ringler et al., 2009, 2013). In short, individuals are identified based on their unique ventral coloration patterns and capture locations mapped on a detailed GIS map (Ringler et al., 2016). Only the capture points in the area where the respective male showed territorial behavior (e.g. calling, courtship, phonotactic approach of a stimulated intruder) were considered for identifying the territories. To avoid disturbance, we did not capture and identify the frogs seen at the pools during the main experiment, but several frogs were captured at the pools during the pilot experiments in 2017, which used similar pool conditions. In addition, some pools from the first transect in 2020 were left in place after 6 days to obtain additional data on how far the frogs traveled. We compiled these opportunistic observations to measure the linear distance from the closest known territory point of each male to the pool where the respective male was captured. Rainfall data were provided by the Nouragues Ecological Research Station from an above-canopy weather station (Nouraflux, rainfall sensor Campbell ARG100).

### Statistical analysis

We assessed whether there were differences between pool conditions in: (1) the probability of tadpole deposition (yes=1, no=0) using a logistic regression by building a generalized linear mixed model (GLMM) with the complementary log–log (cloglog) link function and binomial error distribution; and (2) the number of tadpoles deposited in the artificial pools by building a GLMM with log link function and Poisson error distribution. In both models, pool condition and cumulative rainfall were used as fixed effects, and pool ID was used as an observation-level random effect to model overdispersion in count data (Harrison, 2014). Models were built using the function glmer from the lme4 R package (Bates et al., 2015). Overdispersion for count data was checked using the function dispersion_glmer from the blmeco R package (Korner-Nievergelt et al., 2015). Zero inflation was checked using the function check_zeroinflation implemented in the performance R package (Lüdecke et al., 2021). Likelihood ratio tests were used to determine the best-fit models. The proportion of variance explained by the best-fit models was calculated using the function r.squaredGLMM from the MuMIn R package (https://CRAN.R-project.org/package=MuMIn; Nakagawa and Schielzeth, 2013). Tukey *post hoc* pairwise comparisons were performed with the function glht from the multcomp R package (Hothorn et al., 2008). All statistical analyses were performed in R (https://www.r-project.org/). Data and custom scripts are available from doi:10.6084/m9.figshare.16843480.v2.

## RESULTS

**Fig. 2.**
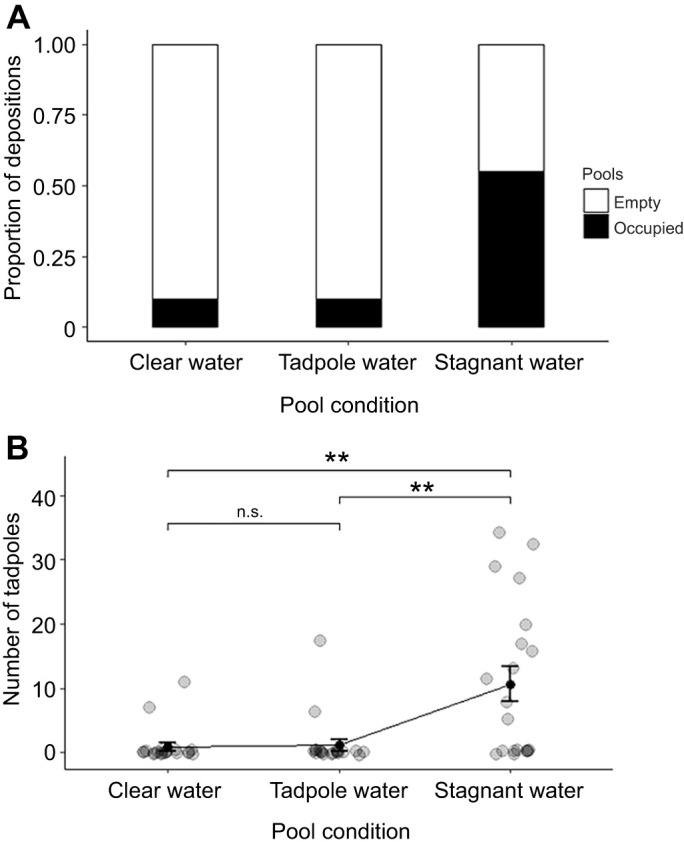
**Poison frogs use stagnant water cues to discover novel breeding sites.** (A) Proportion of occupied pools in each condition after 6 days. (B) Number of *A. femoralis* tadpoles deposited per pool condition after 6 days. The plot depicts the mean number of tadpoles deposited (black circles) and the associated standard errors (whiskers). The grey circles show the number of tadpoles for each pool. **P<0.01.

Of the 60 artificial pools created, 15 pools (25%) were used as deposition sites within 6 days. Most of them (73%) were occupied within the first 2 days. Frogs showed a clear differential pool usage: 11 out of 15 occupied pools were pools from the stagnant water condition (Fig. 2A). Out of the 253 tadpoles deposited, 212 tadpoles were found in the stagnant water condition, 23 in the tadpole water condition and 18 in the control condition. Of the 18 adult frogs observed at the pools (four of which were transporting tadpoles), 15 were at the stagnant water condition. Sixteen frogs captured visiting pools with stagnant water condition had their closest known territory points between 3 and 50 m (median=12 m) from the pools where they were found.

The probability of tadpole deposition was affected by pool condition and cumulative rain (pool condition, X^2^=13.937, d.f.=2, *P*<0.001; rain, X^2^=10.501, d.f.=1, *P*=0.001; Table 1) and fixed effects explained 57.37% of the variance. Pool condition and cumulative rain also had a significant effect on the number of tadpoles deposited in the artificial pools (pool condition, X^2^=13.246, d.f.=2, *P*=0.001; rain, X^2^=8.7149, d.f.=1, *P*=0.003; Table 1) and fixed effects explained 45.41% of the variance. The stagnant water condition had, on average, a higher number of tadpoles per pool (mean±s.e.m.: 10.60±2.73; Fig. 2B), compared with the tadpole water condition (1.15±0.89) and the control condition (0.90±0.64).

**Table 1.**
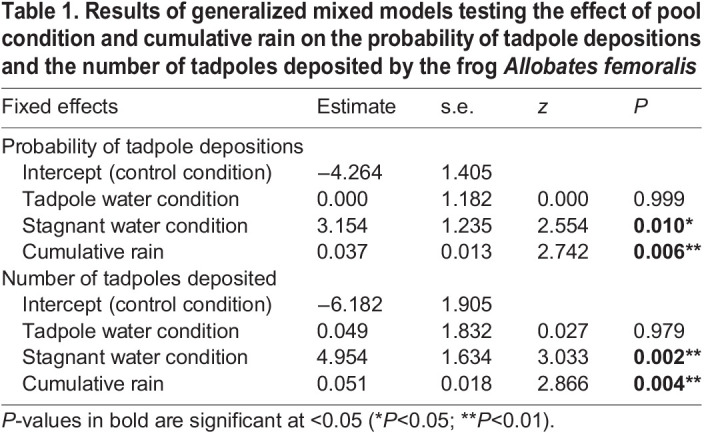
Results of generalized mixed models testing the effect of pool condition and cumulative rain on the probability of tadpole depositions and the number of tadpoles deposited by the frog *Allobates femoralis*

## DISCUSSION

Although water is abundant in the rainforest, suitable water bodies for breeding are rare and difficult to discover by chance. Our findings suggest that frogs rely on olfactory cues associated with stagnant water to find and choose suitable pools and increase their offspring's chances of survival. This discovery provides a better understanding of the mechanisms used by tropical frogs to find new breeding resources in complex and unpredictable environments.

The hypothesis formulated by Pašukonis et al. (2016), that conspecific tadpole odor cues attract *A. femoralis* to novel breeding sites, was not supported by our findings. The presence and size of conspecific tadpoles can be an indicator of habitat permanence (Rojas, 2014; Rudolf and Rödel, 2005) and our results do not exclude the possibility that *A. femoralis* use this information. However, most pools with conspecific tadpoles remained unoccupied after 6 days, suggesting that tadpole odor does not play a major role in the discovery of new pools.

The observed pattern is unlikely to be a result of frogs’ avoidance to deposit in clean water, because frogs readily deposited in the clean water surrounded by olfactory stagnant water cues. In addition, most observed adults and all tadpole carriers were at the stagnant odor pools once again, suggesting that frogs were more likely to discover these pools. Furthermore, most tadpole depositions occurred in the first 2 days after placing the pools, presumably because the odor attracting the frogs decreased as the leaf litter dried out and was washed by rain. Finally, our interpretation is in line with and further supported by tadpole carrier movements observed by Pašukonis et al. (2016), which showed frogs diverting from their normal path to investigate an area where out-of-reach pools with stagnant water and conspecific tadpoles were suspended above the ground. The high number of tadpoles found in the stagnant water condition, together with the observations mentioned above, strongly suggest that our main effect is driven by the pool discoverability rather than a pool preference after discovery. Interestingly, most frogs directly observed at the pools did not carry tadpoles, suggesting that frogs explore and evaluate the pools before transporting tadpoles.

The stagnant odor cues used by frogs to find new breeding sites may also be an indicator of pool stability. Despite having been often noted for temperate-region amphibians (Ogurtsov, 2004; Sinsch, 1990, 2006), the possibility of tropical frogs using olfaction to find new breeding sites and evaluate the basic characteristics of pool stability has been largely overlooked. So far, most studies implicating tropical frog olfaction have focused on detecting predators or conspecifics inside the pools. *Ranitomeya variabilis*, for example, uses olfactory cues to reduce the risk of predation, avoiding pools with cannibalistic conspecific tadpoles (Schulte et al., 2011). *Dendropsophus ebraccatus* detects aquatic egg predators and avoids laying eggs in habitats with fish (Touchon and Worley, 2015). However, very little is known about how frogs evaluate which pools are sufficiently stable for tadpole development. Our study raises a possibility that frogs could use chemical signatures of stagnant water as an indication of pool permanence. Wet decaying leaves, however, are omnipresent in the rainforest, thus the frog-attracting cue must be specific to chemical processes in stagnant water. Future studies should aim to elucidate what chemical signatures present in the stagnant water attract frogs and how they correlate with pool stability.

We recorded frogs arriving to our experimental pools from up to 50 m, but *A. femoralis* and other poison frogs have been shown to find pools and navigate after translocations from hundreds of meters (Pašukonis et al., 2014, 2018, 2019; Ringler et al., 2013). It seems unlikely that odor cues alone could explain accurate navigation over longer distances in the rainforest understory, but in combination with well-developed spatial memory (Beck et al., 2017; Pašukonis et al., 2016), olfaction might be key when exploring new environments. Olfaction has been shown to play an important role in water-finding and navigation in temperate-region amphibians (reviewed in Ferguson, 1971; Ogurtsov, 2004; Sinsch, 1990, 2006), and several authors have suggested that amphibians might imprint on the odor of the natal water bodies (Ogurtsov, 2004; Ogurtsov and Bastakov, 2001; Oldham, 1967; Sinsch, 2014). It is possible that tropical frogs also imprint on the smell that represents suitable breeding sites. For example, a recent study found that *A. femoralis* prefer returning to their natal pools despite the presence of predators, and even when closer pools are available (Ringler et al., 2018). More generally, our results, together with those from other recent studies in poison frogs (Beck et al., 2017; Pašukonis et al., 2016; Schulte et al., 2011), indicate that olfaction might play a crucial but often overlooked role in tropical amphibian spatial behavior and reproduction.

Unpredictable environments such as those of tropical rainforests have driven the evolution of behavioral strategies and sensory abilities, allowing exploitation of small and scattered resources ephemeral in nature. Our study provides the first evidence to suggest that poison frogs use stagnant water cues to discover novel breeding sites. Many tropical frogs rely on small pools for tadpole development; therefore, similar mechanisms could be widespread among tropical amphibians and deserve further investigation.
